# Functional connectivity alterations in the frontoparietal network and sensorimotor network are associated with behavioral heterogeneity in blepharospasm

**DOI:** 10.3389/fneur.2023.1273935

**Published:** 2023-11-09

**Authors:** Xiao-Feng Huang, Xin-Qing Hao, Xiao-Xue Yin, Lu Ren, Da Wang, Feng Jin, Li-Na Tan, Zhan-Hua Liang, Chun-Li Song

**Affiliations:** Department of Neurology, The First Affiliated Hospital of Dalian Medical University, Dalian, China

**Keywords:** blepharospasm, apraxia of eyelid opening, functional near-infrared spectroscopy, frontoparietal control network, sensorimotor network

## Abstract

**Objective:**

Primary blepharospasm (BSP) is a clinically heterogeneous disease that manifests not only as spasmodic closure of the eyelids but also sometimes with apraxia of eyelid opening (AEO). This cross-sectional study aimed to investigate differences in the neural mechanisms of isolated BSP and BSP-associated AEO subtypes, which may reveal the pathophysiology underlying different phenotypes.

**Methods:**

A total of 29 patients manifested as isolated BSP, 17 patients manifested as BSP associated with AEO, and 28 healthy controls underwent resting-state functional near-infrared spectroscopy (fNIRS). We assessed functional connectivity (FC) between regions of interest (ROIs) in the fronto-parietal control network (PFCN) and sensorimotor network (SMN). We also examined the relationship between altered FC and behavioral data.

**Results:**

In the FPCN, ROI- analyses showed decreased FC between the left premotor cortex and supramarginal gyrus in the BSP with AEO group compared to the isolated BSP group. In the SMN, both subgroups showed hypoconnectivity of the left premotor cortex with the right primary motor cortex, primary sensory cortex, and somatosensory association cortex. This hypoconnectivity was positively correlated with the total number of botulinum toxin A treatments, which suggests that long-term botulinum toxin A treatment may modulate motor sequence planning and coordination.

**Conclusion:**

These findings showed different connectivity alterations in neural networks associated with motor and cognitive control among different behavioral phenotypes of BSP. The identification of specific alterations in various networks that correspond to clinical heterogeneity may inform the identification of potential biomarkers for early diagnosis and personalized neuromodulation targets for treating different BSP subphenotypes.

## 1. Introduction

Primary blepharospasm is an adult-onset focal dystonia that is characterized by various types of involuntary overactivation of the periocular muscles leading to partial or total eyelid rim closure (1, 2). The peak age at onset is between the 5th and 7th decades. Excessive involuntary eyelid spasms may lead to functional blindness (2). The pathogenesis of BSP is not fully elucidated, and the most effective treatment is local injections of botulinum toxin (BoNT) into the overactive muscles (3). Although most patients experience significant effects within approximately 2 months, they do not show corresponding improvements in quality of life, especially among BSP patients with AEO (4, 5). Importantly, in recent years, several studies have clearly demonstrated that BSP is a heterogeneous clinical condition that typically presents as forceful spasms of eyelid closure and is often accompanied by AEO, leading to delayed opening of the eyelids or difficulty maintaining open eyelids (6–8). The incidence of AEO in BSP patients may be substantially higher than the typically reported rate of 7% and is especially high (50% ~ 88%) in BSP cases refractory to BoNT-A treatment (9–11). Therefore, a better understanding of the characterized pathophysiological mechanisms of disease heterogeneity may help clinicians develop botulinum toxin injection protocols and identify personalized targets for neuromodulation treatment.

Previous functional neuroimaging studies have demonstrated that BSP is a brain network disorder resulting from the dysfunction of one or more communication nodes within the network (12–14). Several brain regions have been shown to exhibit abnormalities in this disorder, including the sensorimotor cortex, frontal cortex, cerebellum, and brainstem (13–15). However, the results of these studies are inconsistent. In recent years, researchers have increasingly acknowledged that different clinical subphenotypes may represent different underlying pathophysiological mechanisms (2, 16). The findings from a cluster analysis suggested that BSP may be classified into different subtypes according to the type of spasm and the different characteristics of inhibition of the R2 component of the blink reflex recovery cycle (16). Electromyography studies have shown that disruption of the normal reciprocal innervation between the antagonistic levator palpebrae superioris and orbicularis oculi muscles accounts for the occurrence of AEO in BSP patients (17). However, the upstream regulatory mechanism of this disrupted reciprocal inhibition is rarely studied. Only two small-sample positron emission tomography studies have revealed abnormal glucose hypometabolism in the medial frontal cortex and basal ganglia of AEO patients (18, 19). Another reason for the inconsistent results of previous studies may be the different methods used to analyze the activation of local brain regions and the strength of functional connections among brain regions or networks. Recently, increasing attention has been given to the ‘emergent characteristics’ of brain functions; that is, when two brain regions are connected, the functions generated are not localized to any individual brain region, suggesting that brain connections determine the functional organization of the brain (20). Therefore, we believe that it is important to characterize the differences in disruption to brain functional connectivity between patients with isolated BSP and BSP patients with AEO from the perspective of emergent characteristics of brain function (21). Elucidating the differences in brain functional connectivity between the two subphenotypes will not only shed light on the pathophysiological mechanism but also contribute to early differential diagnosis and help clinicians develop personalized treatment strategies. To our knowledge, no studies have yet examined the differences in brain functional connectivity in BSP patients with AEO.

In this fNIRS study, we analyzed the differences in functional connectivity between BSP patients with AEO and patients with isolated BSP. Additionally, relationships between FC measurements and behavioral characteristics (e.g., symptom severity) were evaluated. We hypothesized that network alterations revealed by fNIRS could serve as biomarkers of BSP subtypes (e.g., BSP with AEO).

## 2. Materials and methods

### 2.1. Participants

We recruited patients who were diagnosed with adult-onset primary blepharospasm following the published standard criteria at our outpatient clinic for movement disorders between May 2022 and May 2023 (1, 5). A sudden orbicularis oculi muscle contraction causing eyelid rim narrowing/closure accompanied by eyebrow lowering below the superior orbital margin was classified as an orbicularis oculi spasm, while a delay in reopening the eyelids after involuntary closure without explicit orbicularis oculi contractions with raising of the eyebrows above the superior orbital margin was considered AEO (1). Patients were excluded if they met any of the following criteria: (a) were ≥75 years old; (b) had other forms of dystonia in other locations aside from the upper face (blepharospasm); (c) had received a botulinum toxin injection within 3 months before fNIRS evaluation; (d) showed evidence of traumatic brain injury, dementia, essential tremor, other neurological disorders, or mental disorders; (e) had a history of medication use before the onset of blepharospasm; (f) had a family history of movement disorders; (g) had symptoms too severe to cooperate with the study; or (h) were left-handed. The study was carried out in accordance with the latest version of the Declaration of Helsinki and approved by the ethics committee of the First Affiliated Hospital of Dalian Medical University (No. PJ-KS-KY-2022-253). All participants provided written informed consent. In total, 29 patients exhibited isolated BSP (without AEO or dystonia in other parts of the body), and 17 patients exhibited BSP with AEO; 28 healthy controls were also included in the study. None of the participants used any medications within 24 h before fNIRS evaluation.

The demographic and clinical characteristics, including age, sex, duration of disease, total number of botulinum toxin injections, and average efficacy duration of each botulinum toxin treatment, were obtained from all patients by face-to-face interviews before fNIRS evaluation. Motor symptom severity was evaluated using the Jankovic Rating Scale (JRS). Nonmotor symptom assessments included the Hamilton Anxiety Scale (HAMA), Hamilton Depression Scale (HAMD), and Montreal Cognitive Assessment (MoCA).

### 2.2. Functional near-infrared spectroscopy data acquisition

**Figure 1:**
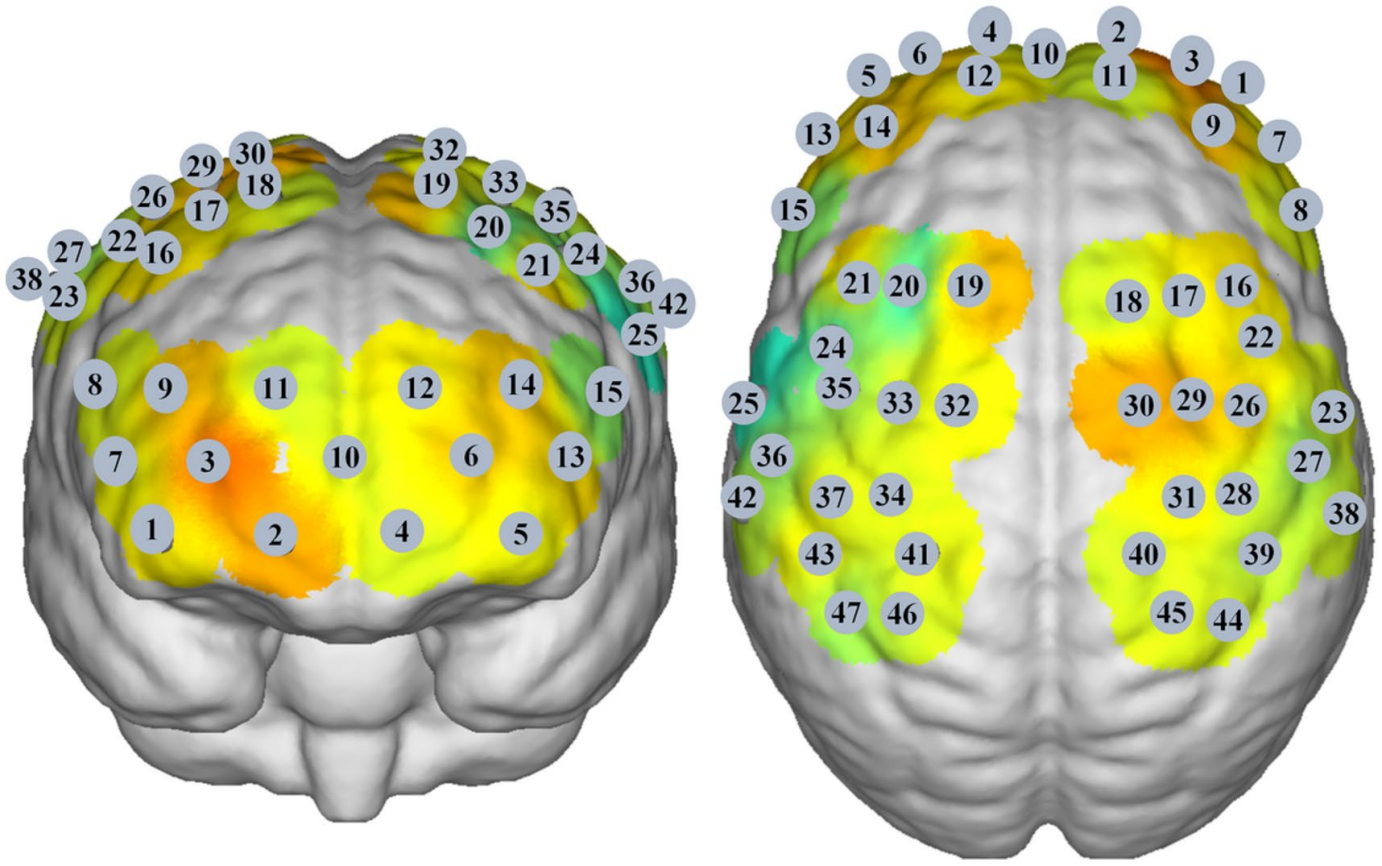
The cortical locations correspond to the channels.

A multichannel near-infrared brain function imaging device (NirSmart, Danyang Huichuang Medical Equipment Co., Ltd., China) was used to record cortical neural activity in the resting state. The emission light sources had wavelengths of 730 nm and 850 nm. There were 23 emitters and 15 detectors, generating 47 effective channels. The distance between channels was 3.0 cm, and the sampling rate of all channels was 11 Hz. The probe set was placed on the scalp above the bilateral prefrontal cortex, parietal lobe, and temporal lobe (Figure 1). NirSpace (Danyang Huichuang Medical Equipment Co., Ltd., China), a three-dimensional positioning system, was used to obtain the Montreal Neurological Institute (MNI) coordinates of the channels. During fNIRS assessment, the participants were seated in a warm, quiet, and slightly dark room. Participants were instructed to sit in a relaxed and comfortable position and to avoid thinking about anything in particular for 5 min.

### 2.3. Data processing

We used NirSpark V0.23.116 (HuiChuang, China) to analyze fNIRS data (22). Data were preprocessed as follows. First, to obtain a stable signal, the first and last 60 s of data were excluded. Then, light intensity was converted to optical density (OD). Then, motion artifacts were corrected (STD threshold = 6, AMP threshold = 0.5). Then, a bandpass filter between 0.01 and 0.2 Hz was applied to remove physiological noise (respiration, cardiac activity, and low-frequency signal drift). Finally, the filtered OD signal was converted to Oxy-Hb and Deoxy-Hb concentrations according to the modified Beer–Lambert law. We used Oxy-Hb as our primary indicator in the following analysis because the Oxy-Hb signal generally has a better signal-to-noise ratio than the Deoxy-Hb signal (23).

Then, we performed FC analysis to detect abnormal patterns of functional connectivity in different BSP patient groups. The changes in Oxy-Hb concentrations at each time point were extracted via the FC-NIRS module of the NirSpark software, and the Pearson correlation coefficients of the time series of Oxy-Hb concentrations of each channel were analyzed (22). The functional connectivity matrix was computed to generate a 47 × 47 correlation matrix for each group (22). Then, we set three pairs of core ROIs in the FPCN, including the bilateral dorsolateral prefrontal cortex (DLPFC) (Ch 16 and 21), bilateral premotor cortex (PMC) (Ch 24, 32, 33, 35, 22, 26, 29 and 30), and bilateral supramarginal gyrus (SMG) (Ch 38 and 47) (24, 25). In addition, we set four pairs of core ROIs in the SMN, including the bilateral PMC (Ch 24, 32, 33, 35, 22, 26, 29 and 30), bilateral primary motor cortex (M1) (Ch 31 and 34), bilateral primary somatosensory cortex (S1) (Ch 27, 39, 40, 36, 37 and 41), and bilateral somatosensory association cortex (S2) (Ch 45 and 46) (26).

### 2.4. Statistical analysis

The demographic and clinical data of the three groups were analyzed using SPSS (IBM SPSS version 25). Sex differences were compared using the χ^2^ test. Comparisons among the three groups were conducted using ANOVAs with *post hoc* Tukey-HSD tests or Kruskal-Wallis ANOVAs with *post hoc* Mann–Whitney U tests. The differences in the duration of disease, JRS score, total number of BoNT-A injections, and duration of BoNT-A efficacy between the two patient groups were compared using the Mann–Whitney U test.

We explored the FC between core areas of the FPCN and SMN using NirSpark (22). One-way analysis of variance was performed to compare FC among the three groups, and Pearson correlation analyses were performed between the time series of each ROI-to-ROI pair. Multiple comparisons were corrected with the false discovery rate (FDR) correction, and *p* < 0.05 was considered to indicate significant differences. In the BSP with AEO group, correlation analyses between behavioral data and abnormal FC were performed using GraphPad Prism 8.0.2, and FDR corrected *p* < 0.05 was considered to indicate significant differences.

## 3. Results

### 3.1. Participants

The BSP with the AEO group had higher JRS scores and a shorter efficacy duration of BoNT-A treatment than the isolated BSP group. In both patient groups, HAMA scores were higher and MoCA scores were lower than those of healthy controls. In addition, we found that AEO symptoms appeared within 1 year of blepharospasm onset in 88% of patients in the BSP with AEO group (Table 1).

**Table 1:** Demographic and clinical characteristics of the participants.

	BSP-aeo (*n* = 17)	Iso-BSP (*n* = 29)	HC (*n* = 28)	Overall *p*-value	*Post-hoc p*-value		
					BSP-aeo vs. iso-BSP	iso-BSP vs. HC	BSP-aeo vs. HC
Age (years)	60.8 (±7.7)	61.0 (±8.5)	57.6 (±6.8)	0.21	–	–	–
Gender M/F	6/11	8/21	9/19	0.85	–	–	–
Duration (months)	51.1 (±57.8)	59.0 (±40.6)	–	0.59	–	–	–
JRS	6.9 (±1.1)	5.2 (±1.3)	–	0.00*	–	–	–
Total times of BoNT-A treatment	7.5 (12)	7.0 (17)	–	0.29	–	–	–
Duration of BoNT-A treatment efficacy	6 (8)	12 (0.5)	–	0.00*	–	–	–
HAMA	6.5 (7)	5 (6)	3 (3)	0.025*	0.754	0.030*	0.023*
HAMD	2 (9)	2 (7)	1 (3)	0.076	–	–	–
MoCA	24 (2)	23 (2)	26.5 (4)	0.008*	0.736	0.004*	0.030*
AEO appeared within 1 year	15 (88%)	–	–	–	–	–	–

Normally distributed variables are shown by Mean ± standard deviations (SD). Non-normally distributed variables are shown by median and interquartile ranges. Iso-BSP, Isolated Blepharospasm; BSP-aeo, Blepharospasm with apraxia of eyelid opening; HC, Healthy Controls; JRS, Jankovic Rating Scale; BoNT-A, botulinum toxin A; HAMA, Hamilton Anxiety Scale; HAMD, Hamilton Depression Scale; MoCA, Montreal Cognitive Assessment; AEO, Apraxia of Eyelid Opening; −, Not Applicable. *p < 0.05.

### 3.2. Between-group differences in functional connectivity

**Figure 2:**
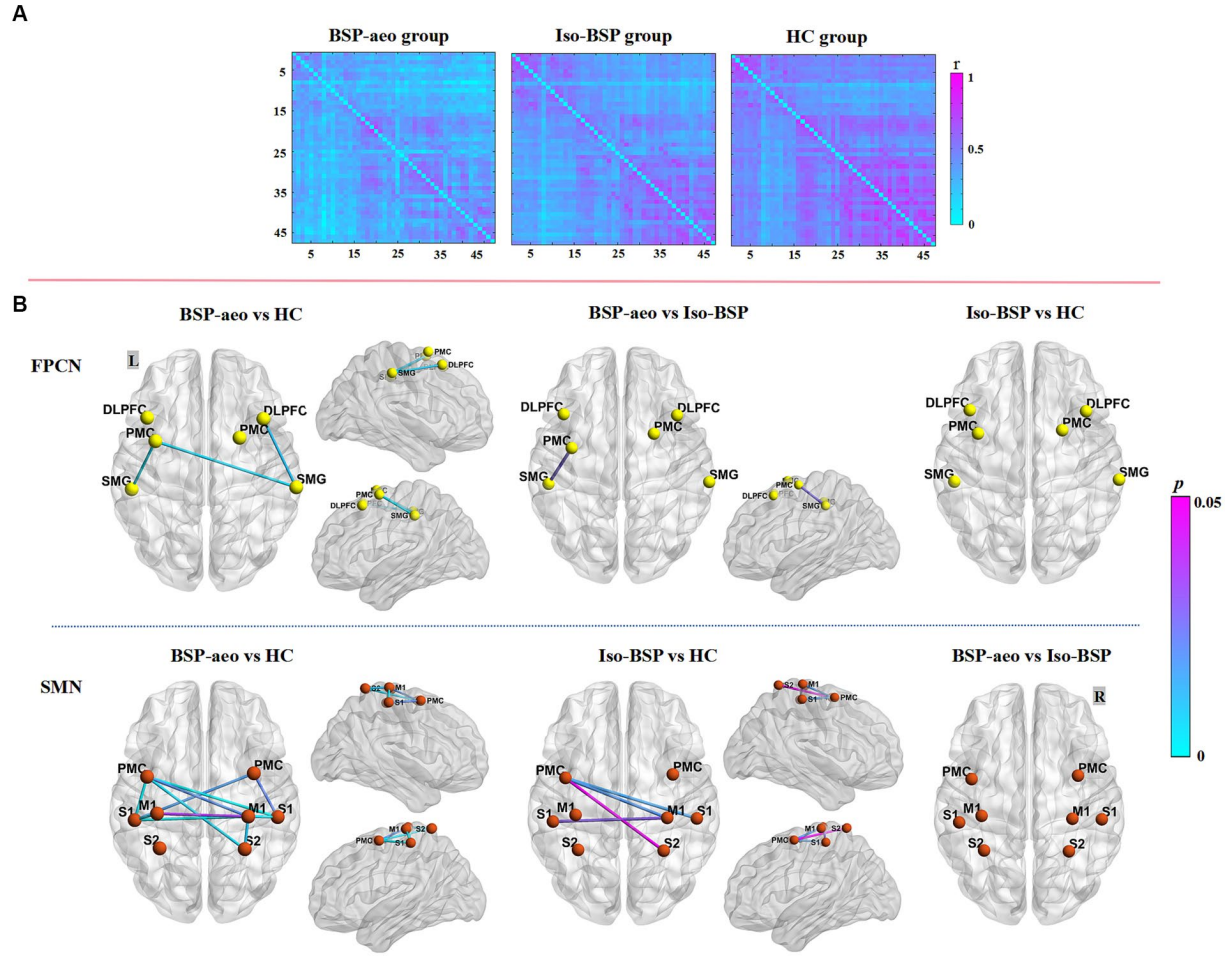
Comparison of functional connectivity between BSP with AEO, isolated BSP, and HC groups. **(A)** The averaged group-level functional connectivity value of all the paired 47 channels within three groups at resting state (as indexed by *r* value correlation matrix). **(B)** Functional connectivity between ROIs among three groups. Regarding FPCN, FC value was more decreased between the left PMC and left SMG in BSP with the AEO group compared to the isolated BSP group, while with SMN, both patient groups showed more decreased FC compared to healthy controls. BSP-aeo, Blepharospasm with apraxia of eyelid opening; Iso-BSP, isolated Blepharospasm; HC, Healthy Controls; ROI, Region Of Interest; *p*-values were FDR-corrected with a set at 0.05. FPCN, Frontoparietal Control Network; SMN, Sensorimotor Network; L, Left; R, Right; PMC, Premotor Cortex; SMG, Supramarginal Gyrus; DLPFC, Dorsolateral Prefrontal Cortex; S1, Primary Somatosensory Cortex; S2, Somatosensory Association Cortex; M1, Primary Motor Cortex.

Figure 2A shows the average FC values of the 47 paired channels at the group level within each group. Different colors represent the different FC values of every paired channel. Figure 2B shows differences in the two networks among the three groups. ANOVAs revealed decreased FC within the FPCN (between the left PMC and left SMG) in the BSP with AEO group compared to the isolated BSP group. Both patient groups showed decreased FC within the SMN compared to healthy controls (Table 2; Figure 2). We did not find significantly increased FC in either patient group compared to healthy controls.

**Table 2:** Functional connectivity differences between patient subgroups and controls and within patient subgroups.

Network	Contrast	ROI 1	ROI 2	FC (*r*)	*p* (FDR-corrected)
FPCN	BSP-aeo < iso-BSP	L. PMC	L. SMG	0.30 ± 0.34 vs. 0.50 ± 0.25	0.004
	BSP-aeo < HC	L. PMC	L. SMG	0.30 ± 0.34 vs. 0.56 ± 0.23	0.004
		L. PMC	R. SMG	0.39 ± 0.38 vs. 0.52 ± 0.31	0.007
		R. DLPFC	R. SMG	0.31 ± 0.30 vs. 0.57 ± 0.19	0.010
	iso-BSP vs. HC	–	–	–	–
SMN	BSP-aeo vs. iso-BSP	–	–	–	–
	BSP-aeo < HC	R. PMC	R. S1	0.46 ± 0.36 vs. 0.70 ± 0.23	0.021
		R. PMC	L. S1	0.37 ± 0.32 vs. 0.63 ± 0.28	0.016
		L. PMC	R. M1	0.52 ± 0.31 vs. 0.78 ± 0.33	0.019
		L. PMC	R. S1	0.44 ± 0.37 vs. 0.78 ± 0.28	0.001
		L. PMC	L. S1	0.43 ± 0.38 vs. 0.78 ± 0.31	0.002
		L. PMC	R. S2	0.42 ± 0.36 vs. 0.75 ± 0.31	0.006
		R. M1	L. M1	0.71 ± 0.37 vs. 0.99 ± 0.46	0.037
		R. M1	R. S1	0.66 ± 0.42 vs. 0.99 ± 0.39	0.005
		R. M1	L. S1	0.45 ± 0.37 vs. 0.87 ± 0.37	0.001
		R. M1	R. S2	0.58 ± 0.45 vs. 0.99 ± 0.45	0.009
		L. M1	L. S1	0.67 ± 0.41 vs. 0.99 ± 0.43	0.004
	iso-BSP < HC	L. PMC	R. M1	0.78 ± 0.33 vs. 0.78 ± 0.33	0.019
		L. PMC	R. S1	0.55 ± 0.27 vs. 0.78 ± 0.28	0.016
		R. M1	L. M1	0.75 ± 0.48 vs. 0.99 ± 0.46	0.036
		R. M1	L. S1	0.62 ± 0.34 vs. 0.87 ± 0.37	0.030

Significant differences in functional connectivity (FC) between the defined group contrasts. *r*-values indicate degree of FC (Mean ± standard deviations). *p*-values were FDR-corrected with a set at 0.05. FPCN, Frontoparietal Control Network; SMN, Sensorimotor Network; BSP-aeo, Blepharospasm with apraxia of eyelid opening; iso-BSP, isolated Blepharospasm; HC, Healthy Controls; ROI, Region Of Interest; L, Left; R, Right; PMC, Premotor Cortex; SMG, Supramarginal Gyrus; DLPFC, Dorsolateral Prefrontal Cortex; S1, Primary Somatosensory Cortex; S2, Somatosensory Association Cortex; M1, Primary Motor Cortex; −, Not significantly different.

### 3.3. Behavioral correlations

In the BSP with AEO group, Spearman correlation analysis showed that higher JRS scores tended to be correlated with decreased FC of the left PMC with the right S1 and S2 within the SMN, but this association was not significant (FDR corrected *p* > 0.05) (Table 3). A higher total number of BoNT-A treatments correlated with stronger FC within the impaired bilateral SMN (including the bilateral PMC, M1, S1 and right S2) and between the left PMC. No correlations of FC values were found with HAMA, HAMD, or MoCA scores or the duration of BoNT-A treatment efficacy.

**Table 3:** Correlations between FC and behavioral measures within BSP with AEO group.

	Network	ROI 1	ROI 2	*r*	*p*	*p* (FDR-corrected)
JRS	SMN	L. PMC	R. S1	−0.5909	0.023	0.069
			R. S2	−0.5444	0.047	0.071
	FPCN	–	–	–	–	
Total times of BoNT-A treatment	SMN	L. PMC	R. M1	0.6743	0.005	0.011*
			R. S2	0.5381	0.049	0.067
		R. PMC	R. S1	0.6462	0.011	0.020*
			L. S1	0.5863	0.024	0.038*
		R. M1	R. S1	0.7557	0.001	0.011*
			R. S2	0.7311	0.004	0.011*
			L. S1	0.7706	0.003	0.011*
		L. M1	L. S1	0.7351	0.003	0.011*
	FPCN	L. PMC	R. SMG	0.5362	0.029	0.087
Duration of BoNT-A treatment efficacy	–	–	–	–	–	
HAMA	–	–	–	–	–	
HAMD	–	–	–	–	–	
MoCA	–	–	–	–	–	

*r*-values indicate the correlation coefficient between behavioral measures and FC. *p*-values were significant < 0.05. JRS, Jankovic Rating Scale; BoNT-A, botulinum toxin A; HAMA, Hamilton Anxiety Scale; HAMD, Hamilton Depression Scale; MoCA, Montreal Cognitive Assessment; ROI, Region of Interest; SMN, sensorimotor network; FPCN, frontoparietal control network; L, Left; R, Right; PMC, Premotor Cortex; S1, Primary Somatosensory Cortex; S2, Somatosensory Association Cortex; M1, Primary Motor Cortex; SMG, Supramarginal Gyrus; −, Not Significantly Correlated; *p < 0.05.

## 4. Discussion

In this study, we used fNIRS to investigate differences in connectivity patterns in BSP patients with AEO, patients with isolated BSP, and healthy controls while controlling for subgroup differences. The results showed that the neural network alterations in the isolated BSP group mainly involved the SMN but not the FPCN. In the BSP with AEO group, in addition to decreased FC within the bilateral SMN, there was decreased FC within the FPCN, mainly involving the prefrontal and posterior parietal cortex. The findings of this study suggest that fNIRS is a valuable method for detecting differences in brain functional connectivity and may help distinguish between the two clinical subphenotypes.

Patients with isolated BSP showed decreased functional connectivity within the SMN, with the left PMC and right M1 especially impacted, which is consistent with previous findings (13, 27). Although BSP is traditionally attributed to basal ganglia dysfunction, the recent understanding of its pathophysiological mechanism emphasizes the abnormal large-scale structural and functional network linking the cortex and subcortical areas (2, 14). The findings from an event-related functional MRI study suggested that blepharospasm patients with reflexive blinks show increased activation in the left M1, right S1, and precuneus (28). It has been reported that the PMC is responsible for initiating voluntary movements, which might be related to the initiation of eyelid opening (29). Thus, functional impairments in the PMC may cause difficulty in the initiation of eyelid opening. Furthermore, decreased FC in the PMC may manifest as a reduction in peripheral inhibition, which leads to excessive muscle contraction and involuntary movement (26, 27). Abnormal brain activity and FC in S1 have been demonstrated in several studies (13, 27, 30). Diffusion tensor tractography also showed abnormal nodal efficiency in S1 (31). Sensory impairment is also an important feature of clinical heterogeneity in BSP, and patients commonly experience pain, a sense of discomfort, an impaired sensory discrimination threshold, and sensory gating (2, 32). As sensory impairment frequently precedes clinical symptoms but involves a wider area than the site of dystonia, it has been considered to be an inherent component of blepharospasm pathophysiology (33). Although the BSP-AEO subtype appears to show more extensive bilateral involvement of the SMN than the isolated BSP subtype, *post hoc* analyses showed no significant differences between the two groups.

Interestingly, the BSP with the AEO group showed alterations in functional connectivity within the FPCN. The FPCN comprises the DLPFC, PMC, and SMG in the posterior parietal cortex and subcortical regions, and the core nodes are the DLPFC and posterior parietal cortex (25, 34, 35). The FPCN has been demonstrated to play a critical role in the selection of relevant stimuli (attentional selection) while building a motor representation and is important in the ability to control, stop, or override motor responses (motor inhibition) (36, 37). Given the critical role of the FPCN in motor inhibition, the impaired FC in the FPCN may account for the central reciprocal inhibition loss of the levator palpebrae superioris and orbicularis oculi muscle pair. Vagefi et al. suggested that deep brain stimulation of the GPi could prevent activation of the levator palpebrae superioris by inhibiting input from the PMC, which promotes AEO (38). Grafman’s theory emphasizes the principles of ‘functional proximity’, whereby higher levels of control necessitate the involvement of more anterior prefrontal regions, and ‘subsidiarity’, whereby regions higher in the hierarchy are recruited only when regions lower in the hierarchy do not provide enough information to control action selection (39). This suggests that, when patients have difficulty opening their eyes due to insufficient activation of the bilateral SMN, the FPCN is recruited proactively for compensation and adjustment of motor signal procedures; however, in BSP with AEO patients, the FC within the FPCN is also impaired, and signals cannot effectively bias and overrule prepotent sensorimotor associations or task sets to optimize controlled action selection (40, 41). Previous studies have shown that the inhibition of a region related to motor planning in cervical dystonia patients showed greater prefrontal and parietal involvement, suggesting that increased input of the more distant prefrontal and parietal cortex compensates for impaired motor planning (42, 43). A previous study reported brain network dysfunction related to the prefrontal cortex in patients with BSP (31). However, FPCN dysfunction has not been studied in previous studies of BSP heterogeneity. The findings of this study indicate that impaired functional connectivity within the FPCN may be a functional neuroimaging biomarker of the BSP with AEO subphenotype. We suggest that the use of fNIRS may facilitate the early identification of the BSP with the AEO subphenotype and the development of more individualized treatment strategies.

Furthermore, in BSP with AEO patients, altered FC within the SMN was positively correlated with the total number of BoNT-A treatments. This suggests that long-term BoNT-A treatment leads to cumulative restoration of disturbed sensorimotor integration. In cervical dystonia, BoNT treatment promoted a shift toward normal brain function in a median nerve somatosensory evoked potential study (44) and decreased abnormal electroencephalography β-band power in the somatosensory-motor cortex (45). However, previous studies have mainly focused on BoNT central effects after 1–1.5 months of injection, which reflects the immediate neuromodulatory effect during peak efficacy but not the long-term effect. Recently, O’Flynn et al. reported that BoNT treatment for 6–12 years resulted in reduced brain activity in the right prefrontal cortex in patients with laryngeal dystonia (46). To the best of our knowledge, this is the first study on the regulatory effect of long-term BoNT treatment for brain functional connectivity in BSP patients with AEO. BoNT was originally believed to block acetylcholine release and prevent the resultant muscle weakness. The mechanisms of BoNT-based central neuromodulation of dystonia pathophysiology are still not well known. It has been speculated that the toxin might be retrogradely transported to the cortex and improve short interval intracortial inhibition (47). However, some researchers believe that the amount of retrogradely transported toxin is likely too small to be clinically effective (48). It is more likely that BoNT decreases the overflow of proprioceptive signaling from the dystonic muscle to the sensorimotor integration network and induces plastic change (48).

There are several limitations to the current study. First, the sample size of the present study was relatively small; thus, the current findings should be considered preliminary, and the results should be confirmed in studies with a larger sample size. Second, because the fNIRS technique measures only some surface brain activity, the activity of the occipital cortex and deep brain structures could not be measured. In addition, the range of ROIs we selected were the corresponding cortical subregions of each channel, rather than the entire functional area.

In summary, BSP patients with AEO showed specific alterations within the FPCN. This specific FC impairment may help researchers better understand the pathophysiological mechanism of disease heterogeneity and facilitate the development of personalized therapeutic and management strategies, such as noninvasive or invasive stimulation technologies targeting the FPCN, which may reduce eyelid opening difficulty.

## Data availability statement

The raw data supporting the conclusions of this article will be made available by the authors, without undue reservation.

## Ethics statement

The studies involving humans were approved by Ethics committee the first affiliated hospital of Dalian Medical University. The studies were conducted in accordance with the local legislation and institutional requirements. The participants provided their written informed consent to participate in this study.

## Author contributions

X-FH: Conceptualization, Methodology, Writing – original draft. X-QH: Formal analysis, Writing – original draft. X-XY: Investigation, Writing – review & editing. LR: Visualization, Writing – review & editing. DW: Investigation, Writing – original draft. FJ: Writing – review & editing. L-NT: Writing – review & editing. Z-HL: Conceptualization, Methodology, Project administration, Writing – review & editing. C-LS: Conceptualization, Methodology, Project administration, Writing – review & editing.
